# A Bayesian analysis of a Test and Vaccinate or Remove study to control bovine tuberculosis in badgers (*Meles meles*)

**DOI:** 10.1371/journal.pone.0246141

**Published:** 2021-01-28

**Authors:** Mark E. Arnold, Emily A. Courcier, Lesley A. Stringer, Carl M. McCormick, Ana V. Pascual-Linaza, Shane F. Collins, Nigel A. Trimble, Tom Ford, Suzan Thompson, David Corbett, Fraser D. Menzies

**Affiliations:** 1 Animal and Plant Health Agency Sutton Bonington, Sutton Bonington, Loughborough, England; 2 Veterinary Epidemiology Unit, Department of Agriculture, Environment and Rural Affairs, Belfast, Northern Ireland; 3 Veterinary Sciences Division, Agri-Food and Biosciences Institute, Stormont, Belfast, Northern Ireland; 4 TVR Field Implementation Unit, Department of Agriculture, Environment and Rural Affairs, Newry, Northern Ireland; The University of Georgia, UNITED STATES

## Abstract

A novel five year Test and Vaccinate or Remove (TVR) wildlife research intervention project in badgers (*Meles meles)* commenced in 2014 in a 100km^2^ area of Northern Ireland. It aimed to increase the evidence base around badgers and bovine TB and help create well-informed and evidence-based strategies to address the issue of cattle-to-cattle spread and spread between cattle and badgers. It involved real-time trap-side testing of captured badgers and vaccinating those that tested negative for bTB (BadgerBCG–BCG Danish 1331) and removal of those that tested bTB positive using the Dual-Path Platform VetTB test (DPP) for cervids (Chembio Diagnostic Systems, Medford, NY USA). Four diagnostic tests were utilised within the study interferon gamma release assay (IGRA), culture (clinical samples and post mortem), DPP using both whole blood and DPP using serum. BCG Sofia (SL222) was used in the final two years because of supply issues with BadgerBCG. Objectives for this study were to evaluate the performance of the DPP in field conditions and whether any trend was apparent in infection prevalence over the study period. A Bayesian latent class model of diagnostic test evaluation in the absence of a gold standard was applied to the data. Temporal variation in the sensitivity of DPP and interferon gamma release assay (IGRA) due to the impact of control measures was investigated using logistic regression and individual variability was assessed. Bayesian latent class analysis estimated DPP with serum to have a sensitivity of 0.58 (95% CrI: 0.40–0.76) and specificity of 0.97 (95% CrI: 0.95–0.98). The DPP with whole blood showed a higher sensitivity (0.69 (95% CrI: 0.48–0.88)) but similar specificity (0.98 (95% Crl: 0.96–0.99)). The change from BCG Danish to BCG Sofia significantly impacted on DPP serum test characteristics. In addition, there was weak evidence of increasing sensitivity of IGRA over time and differences in DPP test sensitivity between adults and cubs. An exponential decline model was an appropriate representation of the infection prevalence over the 5 years, with a starting prevalence of 14% (95% CrI: 0.10–0.20), and an annual reduction of 39.1% (95% CrI: 26.5–50.9). The resulting estimate of infection prevalence in year 5 of the study was 1.9% (95% CrI: 0.8–3.8). These results provide field evidence of a statistically significant reduction in badger TB prevalence supporting a TVR approach to badger intervention. They give confidence in the reliability and reproducibility in the DPP Whole Blood as a real time trap-side diagnostic test for badgers, and describe the effect of vaccination and reduced infection prevalence on test characteristics.

## Introduction

Bovine tuberculosis (bTB) remains an economically important bacterial infection of cattle in the United Kingdom and Ireland [1–4]. The intransigence of bTB to eradication is partially due to the European badger (*Meles meles*) acting as a reservoir host of the causal agent, *Mycobacterium bovis* [5–7]. Arguably, bTB eradication from cattle will require parallel effective control of the infection within wildlife.

bTB control in badgers is complicated by the limitations of established ante mortem diagnostic tests for *M*. *bovis* [8]. Bacteriological culture of swab samples from live animals has a high specificity and comparatively low sensitivity. However it requires sedation of the individual and there is also long interval between sampling and results (~8 weeks) [8]. Blood samples from badgers can be processed using cell mediated tests (e.g. interferon gamma release assay (IGRA)) and serological tests (Dual-Path Platform VetTB test- DPP). IGRA has an estimated sensitivity and specificity of approximately 80.9% and 93.6% using post mortem culture as a gold standard [8, 9]. However this test requires specialised laboratory equipment and testing needs to be carried out within 8 hours of the sample being taken. The DPP VetTB test is based on detection of a serological response to MPB83 or ESAT-6/CFP-10 proteins. The test has been shown to have comparable diagnostic capability as other more established diagnostic tests [10, 11]. Advantages include the possibility of use in the field allowing for real time presumptive diagnosis of infection. Disadvantages include the requirement to be carried out the test at a temperature of between 18°C and 30°C and the DPP has never been validated for use in the field.

Caution is needed in estimating the *M*. *bovis* population prevalence from the proportion of captures testing positive. The likelihood of a positive test is influenced by the characteristics of badgers captured. Male and older badgers are more likely to test positive for *M*. *bovis* [12, 13]. In addition, *M*. *bovis* diseased animals have been hypothesized to be more easily trapped [14]. Difference in trappability have been described with variation attributed to season, age, area and year [15]. A recent study [16] demonstrated how climatic variables alter trappability and found, for example, that drizzle, rain and heavy weather conditions tended to increase trappability, as did minimum temperatures in the range 3–8°C.

To date, bTB control in badgers has mainly involved non-selective culling interventions in England and Ireland [2, 17–20]. Badger vaccination studies have been carried out [21–24] and Ireland is now switching to a vaccination strategy after several years of non-selective culling [25]. A considerable amount of resource is needed to capture badgers, so any intervention ideally requires real time decision making on the fate of each badger at first capture thus negating the need for recapture.

Badger vaccination combined with selective culling presents as an alternative bTB control option. However, there is a current lack of a validated field test that can be utilised at the badger capture site. The availability of such a test would facilitate removal of test positive badgers whilst test negative badgers could be vaccinated and released. This combination of decreasing the burden of infection and enhancing the herd immunity to *M*. *bovis* infection, in theory, could accelerate reduction of the *M*. *bovis* prevalence in a badger population within the area [26]. Moreover, such an approach could dramatically reduce the number of badgers removed compared to the more established proactive culling approach [26].

Tuberculosis vaccines are based on Bacille Calmette–Guérin (BCG) strains of *M*. *bovis* with BadgerBCG being a UK licensed product for use in badgers (Market Authorisation Number 03326/4021) [27]. Unlike natural *M*. *bovis* infection, vaccination with BadgerBCG stimulates very low levels of antibody response to MPB83, CFP10 and ESAT6, which are the main immunodominant antigens found with bTB infection in badgers [28–30]. This difference theoretically enables badgers vaccinated with BadgerBCG to be differentiated from badgers that are naturally infected by a test that detects antibodies to these antigens. The Dual Platform VetTB test (DPP^®^; Chembio Diagnostic Systems Inc., Medford, NY 11763 USA) is such a test, which contains two recombinant antigen (MPB83 and CFP10/ESAT fusion) proteins on an immune-chromatographic strip presented as two separate bands (line 1 and line 2, respectively).

A 5-year field research study investigating a test and vaccination or remove (TVR) regime in badgers was carried out in Northern Ireland with the aim of looking at the feasibility of such an intervention for bTB [31, 32]. This was the first such field study aimed at assessing the viability of using a TVR approach as a future intervention option. Due to market withdrawal of the validated bTB test for badgers, the BrockTB Stat-Pack test (Chembio Diagnostic Systems Inc., Medford, New York, USA), the unvalidated DPP was selected as the field diagnostic test for the project.

During the study, diagnostic samples were obtained from captured badgers for testing by interferon gamma release assay (IGRA) and bacteriological culture as well as DPP testing [32]. This enabled assessment of the annual badger bTB prevalence through Bayesian latent class analysis [33]. This statistical approach also facilitated calculation of the test characteristics of these three diagnostic tests within this population. Additionally, such analyses can also be adapted to measure any changes in test characteristics over the five years of intervention.

The main goal of the analysis presented in this paper was to evaluate the test characteristics of the DPP test (using whole blood as a substrate) and to assess if its performance was adversely affected by carrying out the DPP test under field conditions (compared to a laboratory environment). These results would underpin whether TVR was a practical option for badger intervention in future bTB eradication strategies. A further objective was to use the diagnostic test results obtained over the five years of this study to indicate if there was any statistically significant downward trend in the annual badger bTB prevalence. Furthermore, it was also hypothesized that removal of DPP Field positive badgers would remove a higher proportion of advanced TB cases so the badger population during later years (2016–2018) would have a higher proportion of earlier stage TB infection which may impact on the test results. Therefore, the possibility that sensitivity varied over time due to the impact of the intervention was investigated. The results presented in this paper details the performance of these diagnostic tests over the 5-year study and estimates the annual badger bTB prevalence trend in the area over this time period.

## Materials and methods

### Field work overview

A 5-year (2014–2018) badger intervention study using a selective removal and vaccination regime was undertaken in a 100 km^2^ area of County Down, Northern Ireland. Modelling outputs had suggested that such a time period would be required to enable an effect on badger *M*. *bovis* prevalence to be observed [34]. The TVR Research Project operated under the Animals (Scientific Procedures) Act 1986 (as amended); ASPA Licence Number 2767. Licences were also obtained from Northern Ireland Environment Agency (NIEA) to allow the capture, sampling, and removal of badgers. Each calendar year, badgers were trapped and DPP tested in the field using whole blood (10μl of heparinised blood; DPP Field). The use of whole blood facilitated real time decision making on the fate of each badger. Badgers were removed if they tested DPP Field positive (line 1 and/or line 2 positive) or vaccinated and released if DPP Field negative (both lines negative). This excluded the first year of the study where all badgers were DPP Field tested, vaccinated and released. Badgers were only sampled (under anaesthesia) on first time capture each year, which involved obtaining blood samples and clinical swabs (tracheal aspirate, oropharyngeal swab and from any observed wound swabs) for diagnostic testing. Samples from each badger were also submitted to a laboratory for further DPP and IGRA analysis and for bacteriological culture (swabs and aspirates). A more detailed description of this study is available [31, 32].

Badgers that were found DPP Field negative at each field test were vaccinated by intramuscular injection of BadgerBCG for years 1–3. Due to a global shortage of BCG vaccines, a BCG Sofia strain (Intervax Ltd, Canada) was administered to such badgers during years 4–5.

#### DPP

**Fig 1:**
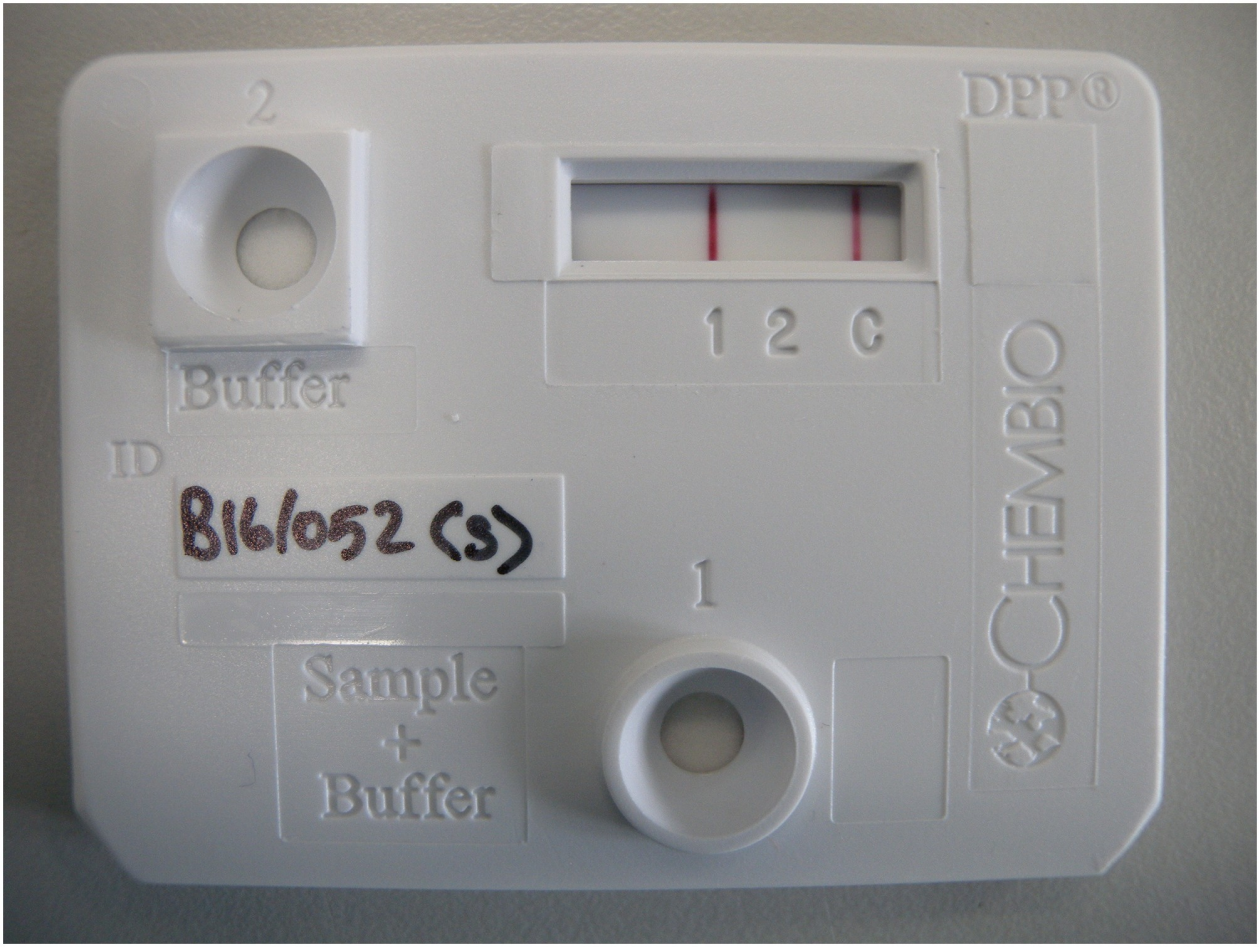
Photograph of DPP test positive at line 1.

For laboratory testing, whole blood (10μl of heparinised blood; DPP Whole Blood) and serum (30μl of serum harvested from clotted blood samples; DPP Serum) were independently analysed using a standard DPP protocol (Chembio Diagnostics Systems Inc., 10-6123-0 Rev 1 November 2012). The result for line 1 and line 2 were separately recorded following visual interpretation with the presence of a visible pink/purple line indicating a positive result (Fig 1). Restricted supplies of DPP test kits limited the number of whole blood samples that were tested during years 1, 4 and 5 (as whole blood samples can only be stored for short intervals; Year 1: 19 badgers (7%), Year 4: 173 badgers(60%), Year 5: 106 badgers (31%)).

#### IGRA

The IGRA enables laboratory testing of heparinised blood samples for cell mediated immune responses to bTB infection through lymphocyte stimulation and subsequent detection of gamma-interferon by ELISA. The protocol used for badger samples was as detailed in [9].

#### Culture

Swabs and tracheal aspirates with individually cultured using standard preparation methods and incubated in liquid BACTEC mycobacterium growth indicator tubes (MGIT) using a Bactec MGIT 960 system (BD Diagnostics, USA) for 56 days (OIE, 2016). Spoligotyping was carried out of any positive cultures to confirm the presence of *M*. *bovis* [35, 36].

### Statistical model

A Bayesian latent class model of diagnostic test evaluation in the absence of a gold standard was applied to the data (See S1 Appendix). For each sample, IGRA, culture and DPP were applied in parallel, either solely DPP Serum (leading to 3 tests applied) or both DPP Serum and DPP Whole Blood (leading to 4 tests). Where both DPP Serum and DPP Whole Blood were included in the model, conditional dependence between these tests was modelled as described in [37]. Given the possibility of interaction with the DPP, badgers that had been previously vaccinated with BCG Sofia (Year 5), were analyzed separately (DPP Whole Blood (BCG Sofia) & DPP Serum (BCG Sofia)).

The initial analysis was focused on the visual interpretation of DPP, and comparison was made between the use of line 1 only, line 2 only or both line 1 and line 2 (badger positive if positive for either line), with the best performing of these used for all subsequent analyses. Comparison was also made between DPP Whole Blood based on laboratory results compared to DPP carried out in the field using whole blood (DPP Field)–see [11].

Informative priors were given for culture specificity (Table 1) but all other priors (covering diagnostic test sensitivity/specificity and infection prevalence each year) were given by beta distributions with both parameters equal to 1 (equivalent to uniform distribution between 0 and 1).

**Table 1:** Prior distributions used for a Bayesian model to estimate the sensitivity and specificity of four diagnostic tests for bovine TB infection in live badgers.

Test	Parameter	Source of prior	Beta prior distribution	Median (2.5^th^– 97.5^th^ percentiles)
Interferon Gamma	Sensitivity (*Se*_*1*_)	Non-informative	(1,1)	
	Specificity (*Sp*_*1*_)	Non-informative	(1,1)	
DPP Whole Blood	Sensitivity (*Se*_*2*_)	Non-informative	(1,1)	
	Specificity (*Sp*_*2*_)	Non-informative	(1,1)	
DPP Serum	Sensitivity (*Se*_*3*_)	Non-informative	(1,1)	
	Specificity (*Sp*_*3*_)	Non-informative	(1,1)	
Culture	Sensitivity (*Se*_*4*_)	Non-informative	(1,1)	
	Specificity (*Sp*_*4*_)	[38]	(1050.8,3.1)	0.997 (0.993–0.999)

Infection prevalence was estimated in the model using two approaches: (i) a different parameter for each year of the study, given an uninformative prior (beta (1,1)), and (ii) representing the prevalence with a declining exponential trend, so that prevalence in year *t* was given by *Aexp(-Bt)*, where *A* and *B* were parameters to be determined. In this trend model, *A* represented the infection prevalence at the start of the study and *B* represented the trend, that is, proportionate annual reduction in badger infection prevalence. *A* was given a uniform prior in the range 0–0.5, a range which was expected to more than cover the possible infection prevalence at the start of the study, and *B* was given a gamma prior with both parameters set to 0.01 (non-informative). These models were compared using the Deviance Information Criterion (DIC) [39], a Bayesian measure of model fit, with the model having the lowest value to be preferred.

All calculations were performed in WinBUGS 3.1, using a burn-in of 5,000 iterations followed by 10,000 iterations of the model. Inspection of the history of each parameter was used to check convergence of individual model runs, and the robustness of the results to different starting values of the parameters was tested by running multiple chains with different starting values and the use of the Gelman-Rubin statistic [40].

Model fit was checked through use of the Bayesian p-value, which is a Bayesian equivalent of a chi square test [41], and compared the observed data with that simulated from the model, using the multinomial cell probabilities with prevalence and test performance based on the posterior densities. This tested whether there was any significant difference between them, with the outcome being a p-value. For the present study, we considered that a p-value of less than 0.05 would indicate that the model was not adequately able to represent the observed data.

It was hypothesized that the intervention may impact on the sensitivity of DPP and IGRA through the disproportionate removal of badgers with more advanced *M*. *bovis* infection. This would mean that a model that allowed the sensitivity of these tests to vary over the course of the study could possibly provide a better fit to the data and a more robust estimate of the infection prevalence. Therefore, the possibility that the sensitivity of DPP and IGRA have varied over time due to the impact of control measures was investigated. This was investigated by allowing the sensitivity of the tests to vary over time, so rather than having a fixed sensitivity for DPP (Whole Blood and Serum) and IGRA over time (in the formula in S1 Appendix), each sensitivity was given the form: logit(Se) = *a*+b*year, where *a* determines the sensitivity at the start of the study (specifically *exp(a+b)/(1+exp(a+b))* gives the sensitivity in the first year of the study) and *b* determines the yearly rate of change. The parameter *b* was of particular importance as it indicated whether there was a significant time-dependent trend. This analysis was carried out with non-informative priors for *a*, *b*, each with normally distributed priors with mean 0 and variance of 100.

## Results

### Summary of diagnostic tests results

The data consisted of 1,519 sets of test results where either 3 or 4 tests were completed in parallel (Table 2). In total, 431 badgers were tested once, 217 twice, 88 three times, 50 four times and 38 five times i.e. in each year of the study; in total there were test results from 824 individual badgers (badgers were tested no more than once per year).

**Table 2:** Summary of the annual number of individual badgers tested for TB and the number that were positive for each test for a test vaccinate remove study data in Northern Ireland for the five years of the study (DPP results were from visual interpretation using line 1 only).

Diagnostic Test	Year				
	1 (%)	2 (%)	3 (%)	4 (%)	5 (%)
IGRA	26/272 (9.6)	22/341 (6.5)	25/271 (9.2)	9/287 (3.1)	4/339 (1.2)
DPP Whole Blood	N/A	32/341 (9.4)	13/271 (4.8)	6/132 (4.5)	1/113 (0.9)
DPP Field	N/A	39/341 (11.4)	9/271 (3.3)	14/287 (4.9)	3/162 (1.9)
DPP Serum	28/272 (10.3)	24/341 (7.0)	11/271 (4.1)	9/287 (3.1)	15/162 (9.3)
DPP Whole Blood (BCG Sofia)	N/A	N/A	N/A	N/A	7/122 (5.7)
DPP Field (BCG Sofia)	N/A	N/A	N/A	N/A	13/179 (7.3)
DPP Serum (BCG Sofia)	N/A	N/A	N/A	N/A	38/179 (21.2)
Culture	11/272 (4.0)	7/341 (2.1)	9/271 (3.3)	2/287 (0.7)	4/339 (1.2)

N/A = not applicable

The relative performance of IGRA compared to DPP was not totally consistent between years, with IGRA having a greater proportion of tests positive than DPP (both Whole Blood (Laboratory and Field interpretations) and Serum) in year 3, but a lower proportion in other years (Table 2). DPP Whole Blood did consistently have a higher proportion of positives than DPP Serum across the four years. Conversely during Year 5, DPP Serum (BCG Sofia) disclosed a higher proportion of positives compared to DPP Whole Blood (BCG Sofia) (Table 2). Overall, there was a similar proportion of animals positive for DPP Whole Blood between the laboratory and field interpretations, with the field interpretation having a slightly higher proportion of samples positive across years 2–5 of the study.

### Visual interpretation of DPP

A preliminary analysis was carried out between the relative performance of DPP (laboratory visual interpretation) using only line 1, only line 2, and both in parallel (positive if either positive for line 1 or line 2) (see Table A in S2 Appendix) for summary data). This indicated that using line 1 only produced the best overall performance of DPP using visual interpretation. Use of line 2 only resulted in very low sensitivity (~14%, Table B in S2 Appendix) and use of both line 1 and line 2 in parallel resulted in very low specificity for DPP Whole Blood (~94%, Table B in S2 Appendix). Subsequently therefore, the results presented in this paper relate only to visual interpretation of DPP line 1 only.

**Fig 2:**
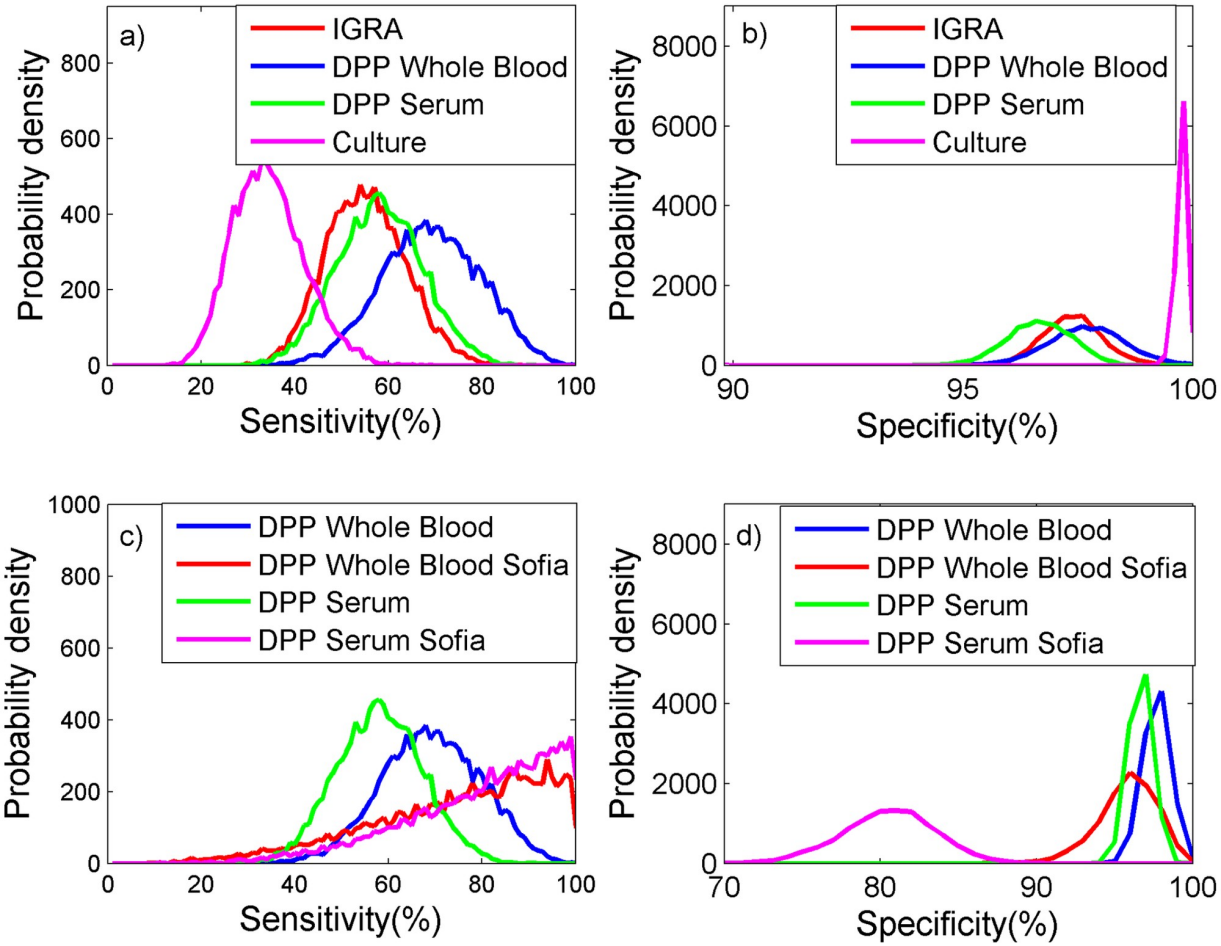
Probability density plots of the performance of 4 tests to detect bovine TB in badgers for a) sensitivity and b) specificity, where DPP was evaluated excluding badgers vaccinated with BCG Sofia and for c) sensitivity and d) specificity, where DPP was evaluated only including badgers vaccinated with BCG Sofia.

Estimates of test performance were similar between IGRA and DPP Serum (Table 3, Fig 2a and 2b), with DPP Serum having slightly higher sensitivity (medians 55% cf. 58%, respectively) and similar specificity (medians 97% cf. 98%, respectively). DPP Whole Blood (laboratory interpretation) was found to higher sensitivity than DPP Serum (medians 69% cf. 58%, respectively) but similar specificity (medians 97% cf. 98%, respectively). Vaccination with BCG Sofia had a significant impact on the estimated sensitivity and specificity of DPP Serum (Fig 2c and 2d), resulting in a higher sensitivity (83%) but a lower specificity (81%) than was observed for DPP Serum from animals not vaccinated with BCG Sofia. However, vaccination with BCG Sofia had comparatively little impact on the performance of DPP Whole Blood. Culture had very high specificity (99.8%) but relatively low sensitivity (34%) compared to the other diagnostic tests used in the study.

**Table 3:** Posterior distributions from a Bayesian model to estimate diagnostic test sensitivity and specificity for bovine TB infection in live badgers (DPP results were from visual interpretation using line 1 only).

Diagnostic Test	Sensitivity		Specificity	
	Median	95% CrI	Median	95% CrI
IGRA	0.55	(0.4, 0.71)	0.97	(0.96, 0.99)
DPP Whole Blood	0.69	(0.48, 0.88)	0.98	(0.96, 0.99)
DPP Field	0.70	(0.47, 0.89)	0.97	(0.95, 0.98)
DPP Serum	0.58	(0.4, 0.76)	0.97	(0.95, 0.98)
DPP Whole Blood (BCG Sofia)	0.78	(0.31, 0.99)	0.96	(0.92, 0.99)
DPP Field (BCG Sofia)	0.43	(0.11, 0.85)	0.93	(0.88, 0.96)
DPP serum (BCG Sofia)	0.83	(0.41, 0.99)	0.81	(0.74, 0.86)
Culture	0.34	(0.21, 0.5)	0.998	(0.995, 1)

CrI = credibility interval

Conditional dependence was found between DPP Whole Blood and DPP Serum, with the sensitivity covariance (see *ρ_Se_* in S1 Appendix) given by (plus 95% credible intervals) 0.009 (0.0006, 0.02) and specificity correlation (see *ρ_Sp_* in S1 Appendix) by 0.14 (0.04, 0.21).

Estimates of the sensitivity and specificity of DPP Field, showed very similar estimates of sensitivity and specificity to DPP Whole Blood (Table 3), with a small (~1% increase in sensitivity for the field test compared to the laboratory result (Table 3), accompanied by a 1% decrease in specificity). For BCG Sofia vaccinated animals, the difference between DPP Field and DPP Whole Blood was much larger, where there was a decrease of both sensitivity and specificity for DPP Field compared to DPP Whole Blood. However, due to there being only one year of data to estimate the sensitivity and specificity of the field interpretation for BCG Sofia vaccinated animals, the credible interval for the sensitivity was very wide for the test applied to these animals, and measures of the difference were not statistically significant (credible interval of the difference included 0 for both sensitivity and specificity).

**Fig 3:**
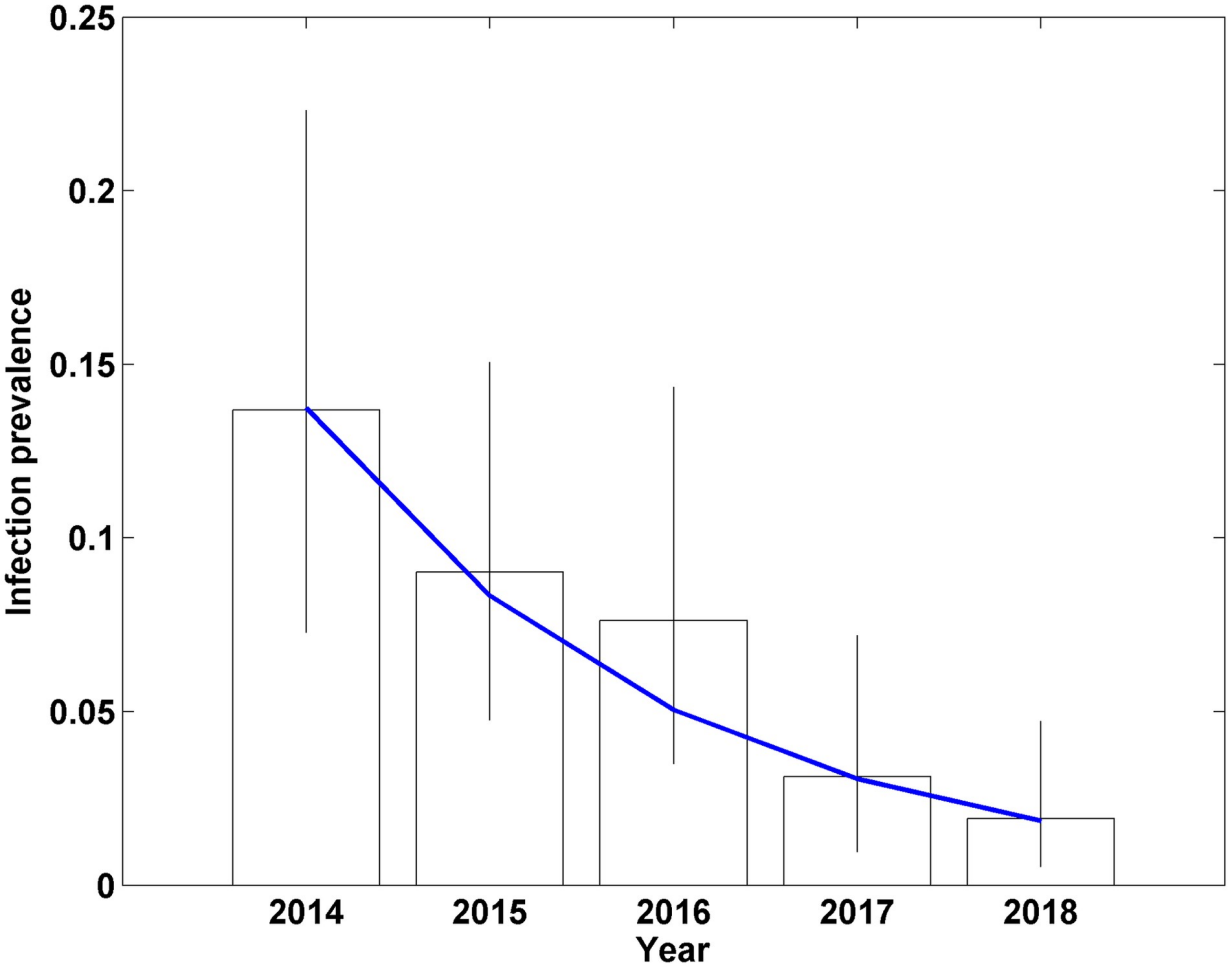
Estimates of bovine TB infection prevalence for badgers in Northern Ireland using a Bayesian model applied to data from 3 diagnostic tests, using independent parameters for the prevalence each year (bars, with vertical lines giving the 95% credible interval of each annual prevalence estimate) and fitting an exponentially declining trend for the prevalence each year (blue line).

Comparison of the DIC between the model where a separate parameter was estimated for infection prevalence each year and that where an exponential model was fitted, showed a lower DIC (difference of 4) for the exponential model, *Aexp(-Bt)* (*A* = 0.14 (95% CrI: 0.10–0.20), *B* = 0.50 (95% CrI: 0.31–0.71)). This suggested an exponential decline model was an appropriate representation of the infection prevalence over the 5 years, with an annual reduction of 39.1% (95% CrI: 26.5–50.9), and an infection prevalence estimate in year 5 of the study of 1.9% (95% CrI: 0.8–3.8). Comparison between the exponential and independently estimated prevalence each year indicated close agreement between the two except for year 3 of the study, where the exponential model had lower prevalence (Fig 3).

### Model fit

Use of the Bayesian p-value, showed no evidence of a lack of model fit, with all values >0.05 for the fit of the model to each data set (grouped by year, whether 3 tests applied or 4, and BCG Sofia vaccination status, (Table C in S2 Appendix)).

#### Varying test sensitivity of DPP and IGRA over time

Models allowing the sensitivity of DPP and IGRA to vary over time did show changes in sensitivity for each of test, with DPP Whole Blood reducing in sensitivity through the study (from 91.9% in year 1 to 36.5% in year 5), and DPP Serum and IGRA increasing (DPP Serum from 57.1% in year 1 to 86.4% in year 5, and IGRA from 46.0% in year 1 to 71.7% in year 5) (Table D in S2 Appendix). However, none of the estimates for parameter *b* were statistically significant for any of the tests (as the 95% credible interval included 0 for each, Table 4), so final results were produced assuming constant test sensitivity (see Table D in S2 Appendix) for the results of varying test sensitivity each year of the study).

**Table 4:** Posterior distributions of parameters determining dependence of diagnostic test sensitivity over time, for a 5 year study of badgers in Northern Ireland, where diagnostic test sensitivity is given by logit(*Se*) = *a*+b*year.

Diagnostic test	Parameters of logit sensitivity (95% CrI)	
	*a*	*b*
DPP Whole Blood	3.84 (0.28, 9.35)	-0.92 (-2.48, 0.44)
DPP Serum	-0.11 (-0.22, 1.14)	0.39 (-0.22, 1.14)
IGRA	-0.4 (-1.84, 1.01)	0.33 (-0.24, 1.24)

## Discussion

Use of Bayesian latent class analysis has become a standard methodology for determining test characteristics of diagnostic assays where there is no gold standard comparator and it also provides an effective method for estimating infection prevalence [33]. In the present study, it has enabled the integration of data from different tests to provide a single estimate of infection prevalence rather than a collection of apparent prevalence figures from the different tests. This is particularly valuable in the area of TB testing, where none of the tests have very high sensitivity.

The DPP is a second generation product that replaced its predecessor, the BrockTB Stat-Pak test (Chembio Diagnostic Systems Inc., Medford, New York, USA). Within the DPP, the antigens are presented in two test strips/lines rather than in one line as with the BrockTB Stat-Pak test. This separation (along with the different independent delivery of the test sample and reagent), may account for the increased DPP test performance over its predecessor both in terms of sensitivity and specificity (BrockTB Stat-Pak test sensitivity estimates 49–58%; specificity estimates 93–97%) [38, 42, 43]. Indeed, these analyses confirm the poor performance of DPP line 2, particularly in relation to specificity, and that only DPP line 1 provided a useful interpretation for bovine TB diagnosis in badgers [11, 44].

The results confirmed the very high specificity of culture of clinical samples. The higher sensitivity observed compared to other studies [38, 43] reported here may be due to the parallel testing of two respiratory clinical samples (tracheal aspirate, oropharyngeal swab) per badger along with wound swabs and/or because of regional factors.

Overall, the DPP performed better than the IGRA, particularly in relation to test sensitivity while the DPP Whole Blood also showed a slightly higher sensitivity than DPP Serum (although there was cross-over between 95% credibility intervals for all these tests). This is consistent with [43] who observed the BrockTB Stat-Pak test to have a slightly higher sensitivity than the IGRA, although their findings differed from previous estimates using the Woodchester Park (WP) badger population [38, 45]. The difference between DDP Whole Blood and DPP Serum results lacks any obvious biological explanation and requires further investigation. Similarly, there was no obvious logistical or badger demography reason why IGRA performance should have been different in Year 3.

The DPP detects serological responses to bovine TB infection, which are thought to occur in animals with more advanced/generalized infection while IGRA detects the earlier cell mediated immune response to mycobacterial infection [46, 47]. At the start of an intervention, one might have expected relatively superior DPP performance as there would be a higher proportion of advanced bTB cases, which would decrease with each annual intervention. The converse would be true for IGRA (Table D in S2 Appendix) and the results would provide some support for this hypothesis although not for DPP Serum estimates. However, caution is required in such interpretations given the wide 95% credibility intervals indicating the study was underpowered to statistically demonstrate any such temporal change.

This difference in reactivity of DPP Whole Blood and DPP Serum was further exacerbated by vaccination of badgers with BCG Sofia, which greatly increased the number of DPP Serum test positives while the number was relatively constant for DPP Whole Blood (and IGRA) in Year 5. Increased DPP reactivity following BCG Sofia vaccination may have been anticipated as it is known to be a relatively high producer of MPB70 (and hence MPB83), which is also the main antigen produced in badger through natural *M*. *bovis* infection and the antigen used in DPP line 1 for antibody detection [29, 30, 48, 49]. The mechanism for DPP Serum being responsive to BCG Sofia vaccination in badgers while it was not observed by DPP Whole Blood requires further investigation. However, this observation suggests that caution is required if BCG Sofia vaccination is to be used in conjunction with DPP selective culling, particularly given the observed reduction in DPP Whole Blood sensitivity in this study.

None of the diagnostic tests appeared to show increased reactivity post-vaccination with BadgerBCG, which is comparable with other findings [11]. Increased attenuation through further genetic deletion has enabled BCG Danish strains to evolve as low producers of MPB70/MPB83 [48]. This adaption facilitates use of DPP as a diagnostic test that differentiates between vaccinated and infected animals (a DIVA test), for which this study and other analyses [11] provide supporting evidence. This would extend to use of the DPP in the field given the favourable DPP Field and DPP Whole Blood comparative results (Table D in S2 Appendix; [11]).

The initial badger TB prevalence (14%) was surprisingly low given that the national badger TB prevalence was estimated to be similar (15.3%; [50]). However, the prevalence is similar to that observed in badgers during the randomized badger culling trial [51]. Nevertheless, even from a relatively low starting prevalence, the model outputs indicated a consistent significant (39%) reduction in badger TB prevalence over the intervention period with a final year estimate of 2% (Fig 2). While it is difficult to apportion this reduction between vaccination and selective culling, other modelling studies would suggest that it is difficult to attribute such benefit from vaccination alone [26]. It is speculated that synergistic effects between both components (selective culling and vaccination) enable results that are comparable to those achieved by proactive culling [26, 34]. Indeed, as with the BrockTB Stat-Pack test, the DPP will tend to remove the more advanced TB cases, which are arguably more infectious [42, 45] while BCG vaccination will protect a proportion of the naive badgers left behind in the area; with vaccine efficacy improving over time at the population level [52, 53]. Badger characteristics such as age and sex have previously been found to influence test positivity [42] through variability in infection prevalence and/or test sensitivity. This may influence the precise estimates of prevalence but is unlikely to amend the main findings of this study. Inclusion of age and sex into the model would adversely affect the statistical power and furthermore, would have no practical application with respect to field implementation of the intervention.

These findings complement those already reported on the field application of the DPP Whole Blood [11] and provide test performance characteristics based on a Northern Ireland badger population. It has validated the use of DPP within the field as a real time diagnostic tool for badgers as well as demonstrating the effectiveness of a test and remove or vaccinate intervention approach in reducing badger TB prevalence.

## Conclusions

This study has provided further validation of the use of DPP Whole Blood as a real time trap-side diagnostic test for badgers, and provides further validation of the use of the DPP Whole Blood, particularly under field conditions. It has also demonstrated that a test and vaccinate or remove approach can significantly reduce badger *M*. *bovis* prevalence (40% per annum), suggesting this option could be considered in future TB control strategies.

## Supporting information

S1 Appendix Description of Bayesian model. (DOCX)

S2 Appendix Additional tables. (DOCX)

S1 Data (XLSX)
