# Supplementary material for: A Bayesian analysis of a Test and Vaccinate or Remove study to control bovine tuberculosis in badgers (Meles meles)
Source: PLoS One. 2021 Jan 28;16(1):e0246141. doi: 10.1371/journal.pone.0246141 (PMC7842978; doi:10.1371/journal.pone.0246141)
Supplement: S1 Appendix — (DOCX) [file pone.0246141.s001.docx]

**A Bayesian analysis of a Test and Vaccinate or Remove study to control bovine tuberculosis in badgers (*Meles meles).***

**S1 Appendix: Description of Bayesian model**

The key step in the latent class model is the calculation of the likelihood of the observed data. For the case where both DPP Whole Blood and DPP Serum were applied in parallel with IGRA and Culture, there are 16 possible outcomes (positive or negative for each of the 4 tests). The probability of each of these outcomes will depend on the overall prevalence of badger infection in year *i* (π_i_), the sensitivity/specificity of each of the tests, denoted by Se_1/_Sp_1,_ Se_2/_Sp_2,_ Se_3/_Sp_3,_ Se_4/_Sp_4_ for IGRA, DPP Whole Blood, DPP Serum and Culture respectively as described in (Branscum, Gardner, Johnson, 2005). The DPP test results with Whole Blood/Serum are possibly correlated, and so conditional dependence is also included, and denoted by $\rho_{Se}, \rho_{Sp}$for the sensitivity/specificity respectively. Denote by P(++++), the probability that IGRA, DPP Whole Blood, DPP Serum and Culture are positive respectively (with similar notation for the other test combinations), P(++++) will equal the probability that the individual badger is infected and detected by all the tests ($\pi Se_{1}\left( Se_{2}Se_{3}+\rho_{Se} \right)*Se_{4}$), plus the probability that the badger is not infected and a false positive for all four tests ($\left( 1-\pi\right)\left( 1-Sp_{1} \right)\left( \left( 1-Sp_{2} \right)\left( 1-Sp_{3} \right)+\rho_{Sp} \right)\left( 1-Sp_{4} \right)$. The full set of outcomes and their respective probabilities are given by:

$$P\left( ++++ \right)=\pi_{i}Se_{1}\left( Se_{2}Se_{3}+\rho_{Se} \right)Se_{4}+(1-\pi_{i})(1-Sp_{1})(\left( 1-Sp_{2} \right)\left( 1-Sp_{3} \right)+\rho_{Sp})(1-Sp_{4})$$

$$P\left( +++- \right)=\pi_{i}Se_{1}\left( Se_{2}Se_{3}+\rho_{Se} \right)\left( 1-Se_{4} \right)+\left( 1-\pi_{i} \right)\left( 1-Sp_{1} \right)\left( \left( 1-Sp_{2} \right)\left( 1-Sp_{3} \right)+\rho_{Sp} \right)Sp_{4}$$

$$P\left( ++-+ \right)=\pi_{i}Se_{1}\left( Se_{2}(1-Se_{3}){-\rho}_{Se} \right)Se_{4}+(1-\pi_{i})(1-Sp_{1})(\left( 1-Sp_{2} \right)Sp_{3}-\rho_{Sp})(1-Sp_{4})$$

$$P\left( ++-- \right)=\pi_{i}Se_{1}\left( Se_{2}\left( 1-Se_{3} \right)-\rho_{Se} \right)\left( 1-Se_{4} \right)+\left( 1-\pi_{i} \right)\left( 1-Sp_{1} \right)\left( \left( 1-Sp_{2} \right)Sp_{3}-\rho_{Sp} \right)Sp_{4}$$

$$P\left( +-++ \right)=\pi_{i}Se_{1}\left( (1-Se_{2})Se_{3}-\rho_{Se} \right)Se_{4}+(1-\pi_{i})(1-Sp_{1})(Sp_{2}\left( 1-Sp_{3} \right)-\rho_{Sp})(1-Sp_{4})$$

$$P\left( +-+- \right)=\pi_{i}Se_{1}\left( \left( 1-Se_{2} \right)Se_{3}-\rho_{Se} \right)(1-Se_{4})+(1-\pi_{i})(1-Sp_{1})(Sp_{2}\left( 1-Sp_{3} \right)-\rho_{Sp})Sp_{4}$$

$$P\left( +--+ \right)=\pi_{i}Se_{1}\left( (1-Se_{2})(1-Se_{3}){+\rho}_{Se} \right)Se_{4}+\left( 1-\pi_{i} \right)\left( 1-Sp_{1} \right)(Sp_{2}Sp_{3}+\rho_{Sp})(1-Sp_{4})$$

$$P\left( +--- \right)=\pi_{i}Se_{1}\left( \left( 1-Se_{2} \right)\left( 1-Se_{3} \right)+\rho_{Se} \right)\left( 1-Se_{4} \right)+\left( 1-\pi_{i} \right)\left( 1-Sp_{1} \right)\left( Sp_{2}Sp_{3}+\rho_{Sp} \right)Sp_{4}$$

$$P\left( -+++ \right)=\pi_{i}(1-Se_{1})\left( Se_{2}Se_{3}+\rho_{Se} \right)Se_{4}+\left( 1-\pi_{i} \right)Sp_{1}\left( \left( 1-Sp_{2} \right)\left( 1-Sp_{3} \right)+\rho_{Sp} \right)(1-Sp_{4})$$

$$P\left( -++- \right)=\pi_{i}(1-Se_{1})\left( Se_{2}Se_{3}+\rho_{Se} \right)\left( 1-Se_{4} \right)+\left( 1-\pi_{i} \right)Sp_{1}\left( \left( 1-Sp_{2} \right)\left( 1-Sp_{3} \right)+\rho_{Sp} \right)Sp_{4}$$

$$P\left( -+-+ \right)=\pi_{i}(1-Se_{1})\left( Se_{2}\left( 1-Se_{3} \right){-\rho}_{Se} \right)Se_{4}+\left( 1-\pi_{i} \right)Sp_{1}(\left( 1-Sp_{2} \right)Sp_{3}-\rho_{Sp})(1-Sp_{4})$$

$$P\left( -+-- \right)=\pi_{i}(1-Se_{1})\left( Se_{2}\left( 1-Se_{3} \right)-\rho_{Se} \right)\left( 1-Se_{4} \right)+\left( 1-\pi_{i} \right)Sp_{1}\left( \left( 1-Sp_{2} \right)Sp_{3}-\rho_{Sp} \right)Sp_{4}$$

$$P\left( --++ \right)=\pi_{i}(1-Se_{1})\left( \left( 1-Se_{2} \right)Se_{3}-\rho_{Se} \right)Se_{4}+\left( 1-\pi_{i} \right)Sp_{1}(Sp_{2}\left( 1-Sp_{3} \right)-\rho_{Sp})(1-Sp_{4})$$

$$P\left( --+- \right)=\pi_{i}(1-Se_{1})\left( \left( 1-Se_{2} \right)Se_{3}{-\rho}_{Se} \right)(1-Se_{4})+\left( 1-\pi_{i} \right)Sp_{1}(Sp_{2}\left( 1-Sp_{3} \right)-\rho_{Sp})Sp_{4}$$

$$P\left( ---+ \right)=\pi_{i}\left( 1-Se_{1} \right)\left( (1-Se_{2})(1-Se_{3)}+\rho_{Se} \right)Se_{4}+\left( 1-\pi_{i} \right)Sp_{1}(Sp_{2}Sp_{3}+\rho_{Sp})(1-Sp_{4})$$

$$P\left( ---- \right)=\pi_{i}(1-Se_{1})\left( (1-Se_{2})(1-Se_{3)}+\rho_{Se} \right)(1-Se_{4})+\left( 1-\pi_{i} \right)Sp_{1}(Sp_{2}Sp_{3}+\rho_{Sp})Sp_{4}$$

ρ_Se_, ρ_Sp_ are sampled from uniform distributions such that the joint probability of the result of both tests is constrained to be between 0 and 1. So, in line with Gardner et al. (2000),

ρ_Se~_uniform((*Se_2_*-1)(1- *Se_3_*), min(*Se_2_*,*Se_3_*) – *Se_2_***Se_3_*))

ρ_Sp~_uniform((*Sp_2_*-1)(1- *Sp_3_*), min(*Sp_2_*,*Sp_3_*) – *Sp_2_***Sp_3_*))

The data were grouped by the number of tests (i.e. 3 or 4), year, and for the last year of the study into BCG Sofia vaccinated and non-BCG Sofia vaccinated animals, with separate multinomial distributions fitted to each. For the parts of the study where there were only 3 tests available (IGRA, DPP Serum, Culture), then there are only 8 possible test outcomes. In this case, the term representing the outcomes for DPP Whole Blood and DPP Serum (Se_2,_ Se_3_) in the above multinomial cell formulae were replaced by *Se_3_*, *1-Se_3_* for the outcome where DPP serum was positive/negative respectively.

As there are 16 possible outcomes, the data arise from a multinomial distribution with the above cell probabilities. This model is fitted to the data using WinBUGS 1.4.

**References**

Branscum, A.J., Gardner, I.A., Johnson, W.O. (2005) Estimation of diagnostic-test sensitivity and specificity through Bayesian modelling. Prev Vet Med 68 145-163.

Gardner IA, Stryhn H, Lind P, Collins MT. Conditional dependence between tests affects the diagnosis and surveillance of animal diseases. Prev Vet Med. 2000;45(1-2):107-22.
