# Supplementary material for: A Bayesian analysis of a Test and Vaccinate or Remove study to control bovine tuberculosis in badgers (Meles meles)
Source: PLoS One. 2021 Jan 28;16(1):e0246141. doi: 10.1371/journal.pone.0246141 (PMC7842978; doi:10.1371/journal.pone.0246141)
Supplement: S2 Appendix — (DOCX) [file pone.0246141.s002.docx]

**S2 Appendix: Additional tables**

**Table A**

**Summary of the annual number of badgers tested/positive for TB using DPP for the Test, Vaccinate, Remove study in Northern Ireland for (i) using line 1 only, (ii) using line 2 only, and (iii) using both lines 1 and 2 (positive if either line is positive).**

| Diagnostic Test | Year | | | | |
| --- | --- | --- | --- | --- | --- |
|  | 1  (%) | 2 (%) | 3  (%) | 4  (%) | 5  (%) |
| DPP Whole Blood  Line 1 | N/A | 32/341 (9.4) | 13/271 (7.6) | 6/114 (5.3) | 1/113 (0.8) |
| DPP Whole Blood  Line 2 | N/A | 27/341 (7.9) | 6/271  (2.2) | 2/114 (1.8) | 0/113  (0) |
| DPP Whole Blood,  Lines 1 and 2 | N/A | 54/341 (15.8) | 17/271  (6.3) | 7/114 (6.1) | 1/113 (0.9) |
| DPP Serum,  Line 1 | 28/272 (10.3) | 24/341  (7) | 11/271  (4.1) | 12/287 (4.2) | 15/162 (9.3) |
| DPP Serum,  Line 2 | 16/272 (5.9) | 6/341 (1.8) | 2/271  (0.7) | 2/287 (0.7) | 4/162 (2.5) |
| DPP Serum,  Lines 1 and 2 | 39/272 (14.3) | 30/341 (8.8) | 11/271  (4.1) | 14/287 (4.9) | 18/162 (11.1) |

N/A = not applicable

**Table B. Posterior distributions from a Bayesian model to estimate diagnostic test sensitivity and specificity of DPP using only line 2 or lines 1 and 2 for detection of bovine TB infection in live badgers.**

| **Diagnostic Test** | **Sensitivity** | | **Specificity** | |
| --- | --- | --- | --- | --- |
|  | **Median** | **95% CrI** | **Median** | **95% CrI** |
| DPP Whole Blood (line 2 only) | 0.14 | (0.05, 0.29) | 0.96 | (0.95, 0.97) |
| DPP Whole Blood (line 1 or 2) | 0.63 | (0.41, 0.85) | 0.94 | (0.92, 0.96) |
| DPP Serum (line 2 only) | 0.59 | (0.36, 0.81) | 0.96 | (0.95, 0.98) |
| DPP Serum (line 1 or 2) | 0.55 | (0.36, 0.77) | 0.97 | (0.95, 0.98) |

CrI = credibility interval

**Table C**

**Summary of Bayesian p-values for the fitting of multinomials to each part of the test data set according to year, whether 3 or 4 tests were applied (i.e. whether DPP Whole Blood tested), and whether badgers were vaccinated with BCG Sofia.**

| Year | Number of tests | BCG Sofia vaccinated | Bayesian P-value of model fit |
| --- | --- | --- | --- |
| 1 | 3 | No | 0.31 |
| 2 | 4 | No | 0.59 |
| 3 | 4 | No | 0.05 |
| 4 | 4 | No | 0.06 |
| 4 | 3 | No | 0.32 |
| 5 | 3 | No | 0.43 |
| 5 | 4 | No | 0.10 |
| 5 | 3 | Yes | 0.28 |
| 5 | 4 | Yes | 0.26 |

**Table D**

**Median estimates of DPP (Whole Blood and Serum) and IGRA test sensitivity estimates from a Bayesian model to estimate diagnostic test sensitivity and specificity for bovine TB infection in live badgers, when fitting a time varying sensitivity to each (CrI = credibility interval).**

| Test | Year | | | | |
| --- | --- | --- | --- | --- | --- |
|  | 1 | 2 | 3 | 4 | 5 |
| DPP Whole Blood  (95% CrI) | 0.95  (0.62, 0.99) | 0.89  (0.63, 0.99) | 0.78  (0.55, 0.95) | 0.55  (0.21, 0.91) | 0.32  (0.02, 0.94) |
| DPP Serum  (95% CrI) | 0.57  (0.36, 0.79) | 0.67  (0.49, 0.84) | 0.75  (0.52, 0.92) | 0.81  (0.51, 0.97) | 0.86  (0.46, 0.99) |
| IGRA  (95% CrI) | 0.49  (0.3, 0.71) | 0.57  (0.4, 0.78) | 0.65  (0.41, 0.91) | 0.72  (0.38, 0.97) | 0.78  (0.34, 0.99) |
|  |  |  |  |  |  |
